# Airway Mycobiota—Microbiota During Pulmonary Exacerbation of Cystic Fibrosis Patients: A Culture and Targeted Sequencing Study

**DOI:** 10.1111/myc.70024

**Published:** 2025-01-16

**Authors:** Cécile Angebault, Louise‐Eva Vandenborght, Laurence Bassinet, Nathalie Wizla, Agnès Ferroni, Rodrigue Dessein, Natacha Remus, Caroline Thumerelle, Nathalie Fauchet, Ralph Epaud, Laurence Delhaes, Françoise Botterel

**Affiliations:** ^1^ Unité de Parasitologie‐Mycologie, Département de Prévention, Diagnostic et Traitement Des Infections CHU Henri Mondor, Assistance Publique Des Hôpitaux de Paris (APHP) Creteil France; ^2^ UR Dynamyc 7380, Faculté de Santé, Univ Paris‐Est Creteil (UPEC), Ecole Nationale Vétérinaire d'Alfort (ENVA); USC Anses, Maisons‐Alfort, France Creteil France; ^3^ GenoScreen Lille France; ^4^ Service de Pneumologie Centre Hospitalier Intercommunal de Creteil Creteil France; ^5^ Service de Gastro‐Entérologie, Hépatologie Pédiatrique, CHU Lille Lille France; ^6^ Service de Microbiologie Clinique CHU Necker‐Enfants Malades, Assistance Publique Des Hôpitaux de Paris (APHP) Paris France; ^7^ Service de Bactériologie CHU Lille Lille France; ^8^ Service de Pédiatrie Générale Centre Hospitalier Intercommunal de Creteil Creteil France; ^9^ Centre Des Maladies Respiratoires Rares CRCM, Respirare Creteil France; ^10^ Service de Pédiatrie CHU Lille Lille France; ^11^ Service de Microbiologie Centre Hospitalier Intercommunal de Creteil Creteil France; ^12^ INSERM, IMRB, Univ Paris Est Creteil Creteil France; ^13^ Service de Parasitologie‐Mycologie CHU Bordeaux, Groupe Hospitalier Pellegrin Bordeaux France; ^14^ INSERM U1045, Université de Bordeaux Bordeaux France

**Keywords:** *Aspergillus*, *Candida*, colonization, cystic fibrosis, internal transcribed spacer, pneumonia, *Scedosporium apiospermum*

## Abstract

**Background:**

The airways of patients with cystic fibrosis (pwCF) harbour complex fungal and bacterial microbiota involved in pulmonary exacerbations (PEx) and requiring antimicrobial treatment. Descriptive studies analysing bacterial and fungal microbiota concomitantly are scarce, especially using both culture and high‐throughput‐sequencing (HTS).

**Objectives:**

We analysed bacterial–fungal microbiota and inter‐kingdom correlations in two French CF centres according to clinical parameters and antimicrobial choices.

**Methods:**

Forty‐eight pwCF with PEx from Creteil (*n* = 24) and Lille (*n* = 24) CF centres were included over 2 years. Sputa were collected for culture and targeted‐HTS (ITS2 and V3‐V4 targets). Sequencing and culture data, along with clinical, radiological and treatment data, were analysed. Two‐level stratified analysis was performed to study potential confounding factors (age, CF mutation, FEV1 and antibiotics) on the centre factor. Inter‐kingdom correlations were analysed.

**Results:**

Significant differences in the bacterial microbiota profile were found between centres (*p*‐value = 0.03). For mycobiota, the taxonomic distribution and diversity were comparable. HTS provided concordant but more detailed information than culture and increased detection of main CF fungi (> 25% more positive samples for *Aspergillus* or *Scedosporium*). FEV1 and systemic antibiotic before PEx influenced bacterial microbiota, but no clinical association was found with the mycobiota. No inter‐kingdom correlation between *Pseudomonas* and fungi was found.

**Conclusions:**

Describing concomitant bacterial and fungal communities of pwCF at the beginning of PEx using culture and HTS shows greater diversity in HTS and better detection in case of low microbial load. Interesting inter‐kingdom correlations were observed, requiring further research on larger cohorts to understand the potential microbial interactions.

## 1 Introduction

Cystic fibrosis (CF) is the most common autosomal recessive genetic disorder often complicated by chronic airway infections [1, 2]. Bacteria, and to a lesser extent, fungi contribute to recurrent pulmonary exacerbations (PEx) requiring anti‐infectious treatments and causing respiratory function decline [1, 3, 4]. Alongside a large amount of bacterial data, a substantial body of data on fungi in CF has been collected through culture‐based studies, national registries or international cohorts [2, 5, 6]. The *Saccharomycotina* subphylum, further called yeasts from the ‘*Candida*’ group, especially *Candida albicans* has been reported as the most prevalent fungi colonising the respiratory tract of patients with CF (pwCF), although their role remains uncertain [2, 7, 8, 9, 10, 11]. Moulds of the *Aspergillus* genus, particularly *Aspergillus fumigatus* , are the second most common fungi in culture and the most commonly involved in PEx [2, 7, 8, 9, 12, 13]. Other fungi, such as *Pseudallescheria/Scedosporium*, *Lomentospora prolificans* or *Rasamsonia* spp. are less common but may also be involved in PEx [14, 15, 16]. Several studies, mainly culture‐based, have investigated factors that influence bacterial and fungal diversity and prevalence in pwCF, including demographic and lifestyle factors, medical conditions, CF centre—or antimicrobial treatments [4, 14, 17, 18, 19, 20, 21, 22, 23]. In recent years, culture‐independent methods based on high‐throughput‐sequencing (HTS) have opened a new field of investigation, allowing the detection of microorganisms that are either uncultivable or present at low levels in the airway of pwCF [24, 25, 26, 27, 28, 29, 30, 31, 32]. Cuthbertson et al. [28] showed that the detection of four major CF fungi (*Candida*, *Aspergillus*, *Scedosporium* and *Exophiala*) was 3 to 20 times higher by HTS than by culture. HTS studies have confirmed the presence of fungal dysbiosis in addition to the bacterial dysbiosis in the lungs of pwCFs and are beginning to reveal the core respiratory mycobiome [25, 28, 30, 33]. Analysis of fungal and bacterial HTS data also allows the investigation of correlations between microorganisms within or between kingdoms [24, 26, 27, 32, 34]. Using targeted‐amplicon HTS (TA‐HTS), Delhaes et al. [26] revealed co‐occurrence patterns between bacteria and fungi with, for example, a co‐occurrence between *Pseudomonas aeruginosa* and uncultivable anaerobes. Similarly, Soret et al. [27] showed an association between *P. aeruginosa* and *Scedosporium apiospermum* which adversely affects the lung function. However, to date, few studies have concomitantly analysed the fungal and bacterial respiratory microbiota in pwCF, using both culture and HTS methods, to study their possible variations in relation to demographic or medical data [24, 35].

Here, we performed a culture and TA‐HTS analysis of fungal and bacterial respiratory microbiota of pwCF hospitalised for PEx in two geographically distinct French CF centres (Lille and Creteil). We aimed to identify shifts in bacterial and/or fungal communities based on demographic and/or medical factors (centre, antibiotic use, spirometry parameters, etc.) using one‐ and two‐level stratified analyses. Additionally, we explored potential inter‐kingdom correlations (co‐occurrence or co‐exclusion between bacteria and fungi) at the time of PEx in pwCF.

## 2 Materials and Methods

### 2.1 Study Population and Sample Collection

Between 2013 and 2015, pwCF admitted to the CRCM (CF Resource Centre) of Lille (Lille Hospital, Hauts de France) and Creteil (Centre‐Hospitalier‐Intercommunal‐Creteil, Ile‐de‐France) for acute PEx [3] were invited to participate in the MucoBacMyco study (*Vaincre la Mucoviscidose*, Grant 2013/2013‐047/03, https://www.vaincrelamuco.org/). Included patients had their sputa collected for routine culture‐based microbial analysis and a part was stored at −80°C for culture‐independent analysis. Patients' information's regarding demographics, past and current medical history at time of PEx as well as drugs used within 3‐months prior PEx and at time of PEx were recorded (Table S1). Patients were followed until the completion of antibiotherapy for PEx.

### 2.2 Ethics

All procedures contributing to this work followed the ethical standards of the Helsinki Declaration revised in 2008. This work was part of the MucoBacMyco project, approved by an Ethics Committee (‘Comité de Protection des Personnes d'Ile de France IX’, N° CPP‐IDF IX‐12‐011). All patients received full information regarding the research project from their physician. If they agreed to participate, they signed informed consent prior inclusion. In case of children, both parents were informed and signed the inform consent.

### 2.3 Statistical Analyses of Participants' Characteristics

Fisher exact and Student *t*‐tests were used for univariate analysis of discrete variables, and Wilcoxon's test for continuous variables (two‐sided tests, significance level = 5%). Factors with *p*‐value*s* < 0.20 were tested via stratified analysis of microbial patterns. Statistics were performed using R software (version 4.3.2, http://www.cran.rproject.org).

### 2.4 Microbial Analyses of the Sputa

As routinely performed, the sputa were inoculated after pre‐treatment with a mucolytic agent and homogenisation by prolonged vortexing onto various agar plates (Chocolate agar‐PolyViteX, 5% horse blood Columbia, 5% sheep blood ANC Columbia, Trypticase soy and Drigalski, bioMérieux, Marcy‐L'Etoile, France) for 2–5 days at 35°C. For fungi, the sputa were inoculated onto Sabouraud‐Chloramphenicol‐Gentamicin slants (Biorad, Marnes‐la‐Coquette, France) and incubated for 21 days at 28°C and 35°C. Additionally, BBL CHROMagar *Candida* plates (Beckton Dickinson, Le‐Pont‐de‐Claix, France) were incubated for 5 days at 35°C. One millilitre of each sputum was aliquoted after homogenization and stored at −80°C for culture‐independent analyses.

### 2.5 DNA Extraction Protocol for High‐Throughput‐Sequencing

Sputa (250 mg) were subjected to mechanical (1.4 mm glass‐beads, two 60 s‐cycles, 6400 rpm, MagNA Lyser, Roche, Mannheim, Germany) and enzymatic lysis (proteinase K). Samples were extracted using DSP DNA midi kit (Qiagen, Hilden, Germany) on QIAsymphony [36] in Creteil. Negative controls (250 μL DNA‐free water) were concomitantly extracted and processed. Extracts were sequenced at GenoScreen (Lille, France).

### 2.6 Library Preparation and Targeted Amplicon High‐Throughput‐Sequencing (TA‐HTS)

Amplicon libraries targeting bacterial V3‐V4 16S and fungal ITS2 regions were prepared in 2017 using the Metabiote protocol (GenoScreen, Lille, France) and sequenced on MiSeq (Reagent Kit V2, Illumina, Evry, France). Three environmental controls (EnvC) consisting of DNA/RNA free water were treated similarly. Artificial bacterial or fungal communities (ABC or AFC, Metabiote, GenoScreen) were used as positive controls.

### 2.7 Taxonomic Assignment, Diversity and Statistical Analysis of Microbial Metagenomics Profiles

Merged ‘pair‐end’ reads were trimmed using Metabiote Online v2.0 pipeline (GenoScreen, Lille). Amplicons with > 97% identity were clustered into operational taxonomic units (OTU, Uclust v1.2.22q) [37]. Bacterial OTUs were assigned to Greengenes 13.8 (www.greengenes.gov) and fungal OTUs to UNITE [38]. Insufficiently annotated OTUs were reassigned using SILVA [39], RDP [40], EzBioCloud (https://www.ezbiocloud.net), Mycobank [41] and FungiBank (https://fungibank.pasteur.fr). Assignments to species and genus levels were accepted with ≥ 98.7% and ≥ 94.5% homology, respectively, and a 0.0 *e*‐value [42].

DESeq2 normalisation was performed using SHAMAN (http://shaman.pasteur.fr) [43, 44]. Shannon, Simpson and Inverse Simpson indexes were computed to compare samples alpha‐diversity for bacterial and fungal microbiota. Principal coordinate analyses (PCoA), calculated with Bray–Curtis matrix/distance, were used to assess between‐samples dissimilarities (beta‐diversity); *p*‐values were calculated using Permanova test. One‐level PCoA was used to study differences between Creteil and Lille's microbial patterns, and two‐level PCoA (= stratified analysis) to examine the impact of confounding factors (age, mutation, Forced expiratory volume in the first second [FEV1], antibiotics, etc.) on the centre factor. The generalised linear model from DESeq2 R package was applied to compare the community structure and taxa abundances between groups. *p*‐values were adjusted according to Benjamini–Hochberg procedure [45]. Inter‐kingdom microbial network analysis was performed using permutation‐renormalization bootstrap method (ReBoot, *n* = 1000 bootstrap replicates) from NetCoMi package (ccrepe function, taxa with < 50 reads were excluded) [46].

TA‐HTS results met expectations for ABC and AFC controls. EnvC taxa were considered as background noise. A non‐parametric Spearman correlation was used to compare EnvC and sample taxonomic distribution. Samples with Spearman‐rank correlation coefficient > 0.7 were excluded.

Raw data are available on Genbank SRA (accession number: PRJNA1011866; https://www.ncbi.nlm.nih.gov/sra/PRJNA1011866).

## 3 Results

### 3.1 Patients' Characteristics

Twenty‐four pwCF participated from each CRCM and five Creteil's patients were < 16 years old. The median age was 24.1 years [8.1–75 years], and the sex‐ratio was 1:1. The median age of CF diagnosis was 6 months [0–66 years]. Eighty‐nine percent (43/48) of patients carried a class II mutation, either alone (*n* = 20, including 16 homozygous F508del) or combined with another class (class I, *n* = 18; class III, *n* = 1, class IV, *n* = 3; class IV, *n* = 5). Two patients carried only a class I mutation and data were missing for three others. The most common non‐F508del mutations were G542X (4/48) and A2183G, G85E, R553X, G2789A and S434X (2/48, each). The major CF‐related disorders included exocrine pancreatic insufficiency (94%, 45/48), respiratory disorders (73%, 35/48), nasosinusal diseases (35%, 17/48) and diabetes (23%, 11/48). The two groups, which differ in terms of geographical area, were similar in all characteristics but the mutations rate. Indeed, 50% of Creteil patients had homozygous F508del mutation, compared with 17% in Lille (*p*‐value = 0.03). The characteristics of the patients are presented in Table 1.

**TABLE 1:** Demographics and medical history of the study 48 cystic fibrosis patients according to their geographic area.

	*N* = 48 (%)	Creteil, *n* = 24 (%)	Lille, *n* = 24 (%)	Unadjusted *p*‐value
Median age (y) [range]	24.1 [8.1–75]	21.7 [8.1–51.6]	26 [18–75]	NS
Sex ratio M/F	1	0.8	1.2	NS
Median age at diagnosis [range]	0.5 [0–66]	1.5 [0–42]	0^a^ [0–66]	NS
CF mutations^b^				
Class I	2	1	1	0.03
Class I‐II	18	7	11	
Class II	20	14	6	
Class II‐III	1	0	1	
Class II‐IV	3	2	1	
Class II‐V	1	0	1	
Respiratory diseases	35 (72.9)	18 (75)	17 (70.1)	NS
Bronchiectasis	17 (35.4)	12 (50)	5 (20.8)	
Allergic bronchopulmonary aspergillosis	16 (33.3)	7 (29.1)	9 (37.5)	
Asthma	8 (16.7)	6 (25)	2 (0.08)	
Nasosinusal polyposis	15 (31.3)	8 (33.3)	7 (29.1)	NS
Exocrine pancreatic insufficiency	39 (81.2)	21 (87.5)	18 (75)	NS
Diabetes	11 (22.9)	6 (25)	5 (20.8)	NS
BMI < 18	11 (22.9)	7 (29.1)	4 (16.6)	NS
Median FVC (%) [range]	68 [25‐109]^c^	74 [38–109]	59 [25‐91]^c^	0.01
Median FEV1 (%) [range]	50 [20‐92]^c^	61.5 [27–92]	40 [20‐71]^c^	< 0.01

Abbreviations: BMI, body mass index; CF, cystic fibrosis; FEV1, Forced expiratory volume in the first second; FVC, Forced vital capacity.

^a^ Four missing data.

^b^ Three missing data among Lille patients.

^c^ One missing data.

Eight patients (Lille, *n* = 2; Creteil, *n* = 6) were included twice at more than 6 months interval, and 56 PExs (Lille, *n* = 26; Creteil, *n* = 30) were analysed. In the year preceding PEx, patients had six medical visits on average ([0–13]; Creteil, *n* = 5 [0–12]; Lille, *n* = 6 [2, 3, 4, 5, 6, 7, 8, 9, 10, 11, 12, 13]; NS), one hospitalisation ([0–8]; Creteil, *n* = 1 [0–8]; Lille, *n* = 1 [0–5]; NS) with a median stay length of 2 days [0–43]. Four pwCF (7%) received long‐term oxygen therapy (Creteil, *n* = 3, Lille, *n* = 1; NS). Pre‐PEx treatments and medical characteristics at time of PEx are described in Table 2; and Table S2.

**TABLE 2:** Treatments in the year prior to the pulmonary exacerbation (PEx) and medical examination at time of PEx.

	All PEx, *n* = 56 (%)	Creteil, *n* = 30 (%)	Lille, *n* = 26 (%)	Unadjusted *p*‐value
Treatments within 3 months prior to PEx				
Inhaled antibiotics	38^b^ (70.3)	18^a^ (62.1)	20^a^ (80)	NS
Colistin		8	12	
Tobramycin		6	7	
Aztreonam		—	1	
Colistine + tobramycin (+ aztreonam)		3 (+1)	—	
Inhaled antifungal drugs	2^a^ (3.6)	0 (0)	2^a^ (8)	NS
Posaconazole/voriconazole			1/1	
Systemic antibiotics	29^a^ (52.7)	20 (66.6)	9^a^ (36)	0.03
1 course		12	3	0.03
2/3 courses		6/—	5/1	NS
Antibiotic cycling (aminopenicillin/1st gen. cephalosporin ± macrolide)^h^, ^i^		2	—	NS
Narrow/mid‐spectrum antibiotics targeting MSSA and *H. influenzae*	19^a^ (34.5)	15 (50)	4^a^ (16)	0.01
Aminopenicillin (amoxicillin, amoxicillin/clavulanate)		12	—	< 0.001
Clindamycin/co‐trimoxazole/ciprofloxacin		1/1/1	—/1/3	NS
Extended spectrum and anti‐MRSA antibiotics	20^a^ (36.3)	9 (30)	11^a^ (44)	NS
Monotherapy: Linezolid/ceftriaxone		—	3/1	
GNB‐ESBT (anti‐*Pseudomonas* ± anti‐*Stenotrophomonas*)		8	7	
GNB‐ESBT ± anti‐MRSA		1^j^	1^k^	
Systemic antifungal drugs^h^	4^a^ (7.2)	2 (6.6)	2^a^ (8)	NS
Itraconazole/posaconazole		1/1	2/0	
Long‐term sub‐clinical azithromycin	27^b^ (50)	18^a^ (62.1)	9^a^ (36)	NS
Inhaled bronchodilators	37 (66.1)	16 (53.3)	21 (80.7)	0.05
Inhaled dornase alpha	26 (46.4)	7 (23.3)	19 (73.1)	< 0.01
Inhaled steroids	28 (50)	10 (33.3)	18 (69.2)	< 0.01
Systemic steroids	4 (0.1)	2 (0.07)	2 (0.08)	NS
Medical examination at time of PEx				
BMI < 18	14^a^ (25.5)	9^a^ (31.0)	5 (19.2)	NS
Persistent cough	31^a^ (56.3)	21 (0.7)	10^a^ (40)	
Dyspnea	35^g^ (71.4)	14^f^ (58.3)	21^a^ (84)	
Nasosinusal symptoms	16^a^ (29.1)	5^a^ (17.2)	11^a^ (44)	
O_2_ saturation < 95%	13^g^ (27.7)	8^e^ (32)	5^d^ (22.7)	
Systemic antibiotics	8^c^	6	2	NS
Lung function at time of PEx				
Median FVC (%) [range]	67.5 [25–109]^b^	73 [38–109]^a^	59 [25–91]^a^	0.01
Median FEV1 (%) [range]	48 [20–92]^b^	60 [25–92]^a^	37 [20–75]^a^	< 0.01

*Note:* A detailed description of GNB‐ESBT combinations can be found in Table S2.

Abbreviations: BMI, body mass index; ESBT, extended spectrum bi/tritherapy; FEV1, Forced expiratory volume in the first second; FVC, Forced vital capacity; gen., generation; GNB, Gram‐negative bacteria; *H. influenzae*, *Haemophilus influenzae*; MRSA, methicillin‐resistant *Staphylococcus aureus* ; MSSA, methicillin‐sensitive *Staphylococcus aureus* ; O_2_, oxygen; PEx, pulmonary exacerbation.

^a^ One missing data.

^b^ Two missing data.

^c^ Three missing data.

^d^ Four missing data.

^e^ Five missing data.

^f^ Six missing data.

^g^ Nine missing data.

^h^ Antibiotic cycling and systemic antifungal drugs were given for prolonged treatments (< 3 m).

^i^ Patients receiving antibiotic cycling did not receive additional antibiotic courses.

^j^ Anti‐MRSA = vancomycin.

^k^ Anti‐MRSA = linezolid.

The main PEx symptoms were dyspnea (71%), persistent cough (56%) and O2 saturation below 95% (28%). The body mass index (BMI) was < 18 in 14/55 pwCF (25%). Forced vital capacity (FVC) at inclusion was 67.5% [25%–109%] and FEV1 was 48% [20%–92%] [2]. Both were significantly different between Creteil and Lille (*p*‐value = 0.001 for FVC and 0.01 for FEV1, Table 2), where Lille's patient having more impaired respiratory function. At the time of PEx, eight patients were receiving systemic antibiotics (cefadroxil, *n* = 1; amoxicilline ± clavulanate acid, *n* = 3; aztreonam + amoxicillin/clavulanate, moxifloxacin, linezolid, or meropenem, *n* = 1 each).

Within the 3 months preceding PEx, 70% of patients had received inhaled antibiotics (mainly colistin and tobramycin), and 53% received systemic antibiotics (one course, *n* = 15; ≥ 1 course, *n* = 12). The most frequently used were aminopenicillin targeting *H. influenzae* and/or methicillin‐sensitive *Staphylococcus aureus* (MSSA) (*n* = 12, 22%) and extended spectrum bi/tritherapy (ESBT) targeting Gram‐negative bacteria (GNB, *n* = 15, 27%). The use of systemic, but not inhaled, antibiotics prior to PEx was significantly higher in Creteil (66.6% vs. 36%, *p*‐value = 0.03), mainly single course (40% vs. 12%, *p*‐value = 0.03) of aminopenicillin (12/30 vs. 0/26, *p*‐value < 0.001) compared to Lille CRCM management. Equal number of patients received 2–3 antibiotic courses in both centres (20% vs. 24%). Three Creteil's patients were on long‐term antibiotics (> 3 months, meropenem [*n* = 1] or antibiotic cycling [*n* = 2]), none in Lille CRCM (*p*‐value = NS). Additionally, half of the patients received long‐term sub‐clinical azithromycin, less than 10% were on inhaled (3.6%) or systemic (7.2%) antifungals, 50% on inhaled steroids and 0.1% on systemic steroids. Significantly more patients from Lille were on inhaled bronchodilators, dornase alpha and steroids compared to Creteil (81% vs. 53%, *p*‐values = 0.05; 73% vs. 23%, *p*‐value < 0.01 and 69% vs. 33%, *p*‐value < 0.01, respectively).

### 3.2 Culture, 16S and ITS2 TA‐HTS Results

**FIGURE 1:**
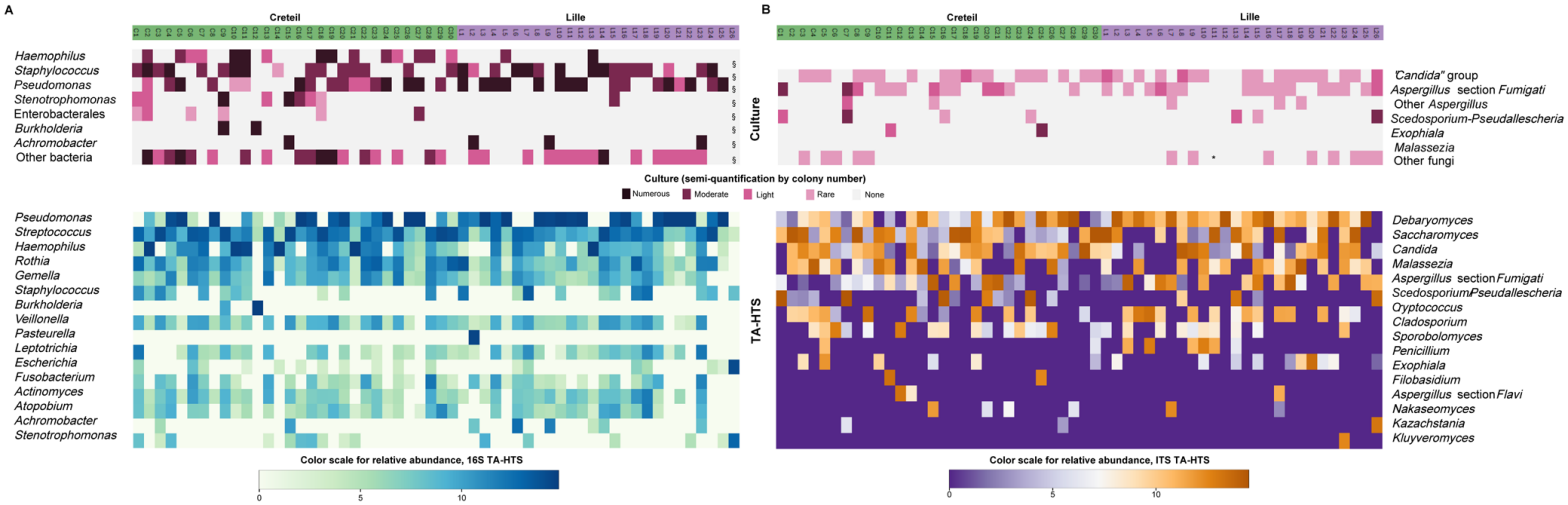
Combined heat map of the bacterial (A) and fungal (B) relative abundances of the predominant taxa detected through culture and targeted‐amplicon high‐throughput‐sequencing (TA‐HTS). §Missing data for the bacterial culture; *detection of *Pneumocystis jirovecii* by qPCR. Patients from Creteil are marked in green, those from Lille in purple. For bacterial culture, semi‐quantification is distributed as follows: rare: < 10^3^ UCF/mL; light: 10^3^–10^4^ UFC/mL; moderate: 10^5^–10^6^ UFC/mL; numerous: ≥ 10^7^ UFC/mL. For fungal culture, semi‐quantification is distributed as follows: rare: 1–10 colonies; light: 10–50 colonies; moderate: 50–100 colonies; numerous: ≥ 100 colonies. For 16S‐HTS and ITS2‐HTS, the relative abundances of the 16 most predominant taxa are represented in the heat map. The concordance between the culture and TA‐HTS for the main CF microorganisms is detailed in Figure S1.

All (55/55, data missing for one patient) bacterial cultures of sputa were positive and 91% of pwCF (50/55) carried at least one pathogenic bacterium in pathogenic quantities (Figure 1A; [47]). Overall, 60% (33/55) of sputa were positive for *Pseudomonas aeruginosa* ; 56% (31/55) for *Staphylococcus aureus* (51% with ≥ 10^5^ UFC/mL); 31% (17/55) for *Haemophilus* sp. (9% with ≥ 10^7^ UFC/mL); 16% (9/55) for *Stenotrophomonas maltophilia* (7% with ≥ 10^5^ UFC/mL); 11% (6/6) for Enterobacterales (< 10^7^ UFC/mL); 7% (4/55) for *Achromobacter xylosoxidans* (with ≥ 10^5^ UFC/mL); 3% (2/55) for *Burkhoderia* sp. No *Streptococcus pneumoniae* and *Moraxella catarrhalis* were detected in the samples. For fungi, the culture was positive in 89% (53/56) of sputa (Figure 1B). Sixty‐two percent (35/56) of pwCF were positive for yeasts of the ‘*Candida*’ group (*Saccharomycotina* yeasts, mainly *Candida albicans* , 44.6%); 51% (29/56) for *A. fumigatus* ; 9% (5/56) for other *Aspergillus* sp.; 12.5% (7/56) for *Scedosporium/Pseudallescheria* sp.; 3.5% (2/56) for *Exophiala* sp. and 23% for other fungi (including *Penicillium* sp., *Cladosporium* sp., *Geotrichum* sp., *Fusarium* spp., *Rhizomucor* sp. and *P. jirovecii*).

After trimming of TA‐HTS data, we obtained 1,922,814 and 1,332,383 reads (median: 42,204 [25,332–55,539] and 26,011 [9302–49,762] reads/sample) and 428 and 200 OTUs (median of 36 and 10 OTUs/sample) for 16S and ITS2, respectively. At the genus level, bacterial OTUs were classified into 71 taxa (median of 19 taxa/sample [3–43]) and fungal OTUs into 88 taxa (median of 7 taxa/sample [2–16]).

**FIGURE 2:**
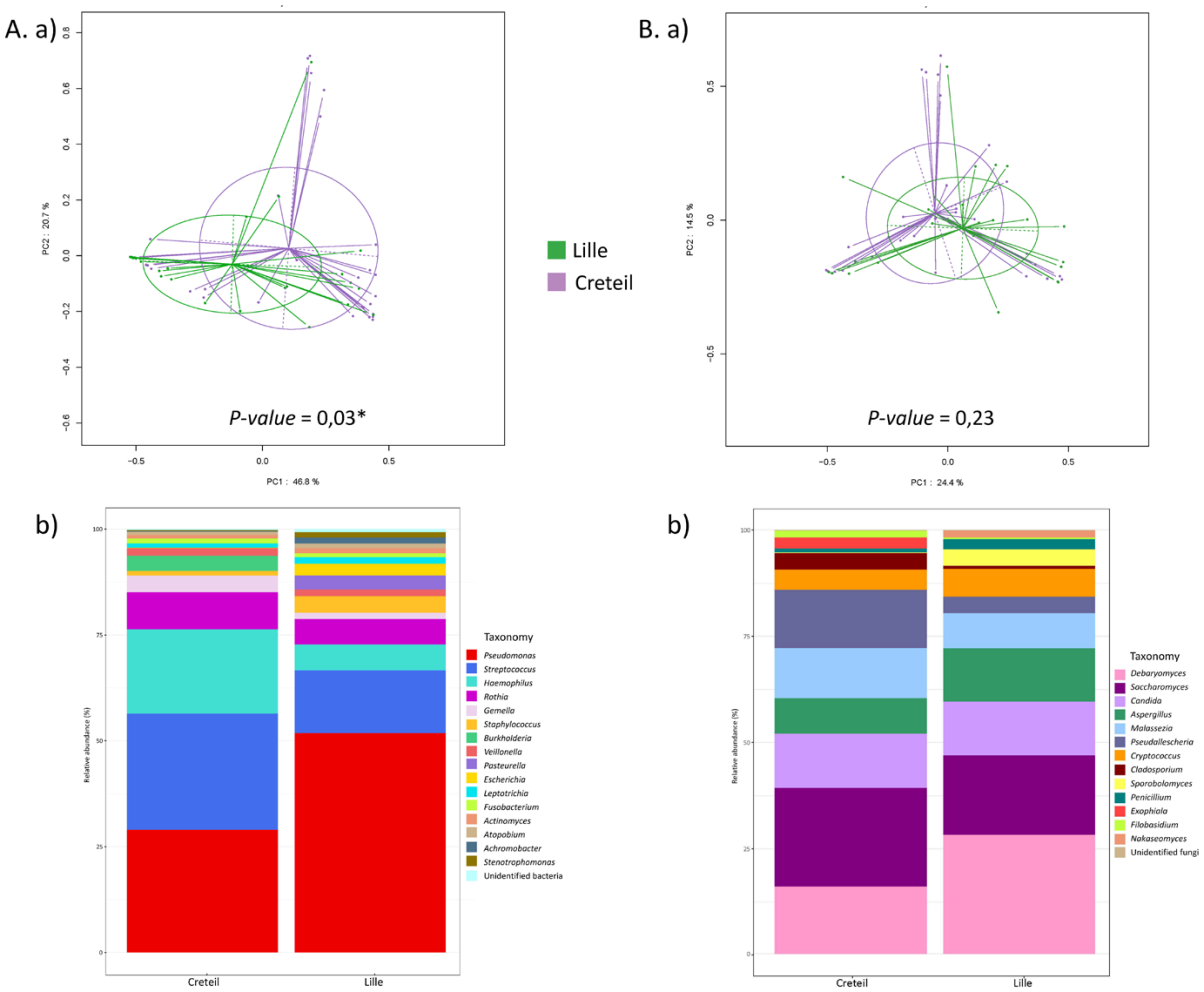
Diversity of bacterial and fungal microbiota in sputa of CF patients at time of pulmonary exacerbation (PEx) according to their centre (Creteil or Lille). Thirty sputa were collected from Creteil's CF patients and 26 from Lille's. (a) Beta‐diversity of bacterial (A) or fungal (B) microbiota assessed via PCoA analysis, statistical significance was assessed using Permanova test. (b) Taxa plots summarising the relative abundances of most predominant bacterial (A) or fungal (B) genera identified by centre. The diversity indexes (alpha‐diversity measurements) for bacterial and fungal metagenomic analyses are available in Table S3.

Predominant bacterial taxa were *Pseudomonas*, *Streptococcus*, *Haemophilus* and *Rothia* accounting for 38.4%, 20.9%, 13.1% and 7.3% of bacterial reads, respectively (Figure 2A). These taxa belonged to the core microbiome with a relative abundance of > 1% in > 50% of the samples. *Gemella*, *Staphylococcus*, *Veillonella* and *Actinomyces* were less represented (2.7%, 2.3%, 1.7% and 0.9% of all reads, respectively) albeit with > 1% relative abundance in > 20% of the samples.

Regarding fungi, *Debaryomyces*, *Saccharomyces*, *Candida*, *Aspergillus*, *Malassezia*, *Pseudallescheria* and *Cryptococcus* were predominant with 19.9%, 19.6%, 11.8%, 9.6%, 9.4%, 8.5% and 5.1% of fungal reads, respectively (Figure 2B). *Debaryomyces*, *Saccharomyces*, *Candida* and *Malassezia* were part of the core microbiome (> 1% relative abundance in > 50% of the samples). *Cryptococcus* and *Pseudallescheria* had a relative abundance of > 1% in > 15% of the samples.

The concordance between culture and TA‐HTS for the main CF bacterial taxa was above 30%. Concordance of 100% (2/2) for *Burkholderia* sp.; 72% (28/39) for *Pseudomonas* sp.; 55% (19/34) for *S. aureus* ; 50% (4/8) for *Achromobacter* sp.; 39% (7/18) for *Stenotrophomonas* sp. and 35% (16/46) for *Haemophilus* sp. (Figure S1). For the main CF fungi, the concordance was above 26% with 100% for *Exophiala* sp. (2/2); 62% (35/56) for yeasts of the ‘*Candida*’ group; 63% (29/46) for *A. fumigatus* ; 41% (7/17) for *Scedosporium/Pseudallescheria* spp. and 27% (4/15) for other *Aspergillus* spp. (Figure SA). *Malassezia* spp. were detected only by HTS (*n* = 33).

### 3.3 Fungal–Bacterial Correlations in CF Patients at Time of Exacerbation

**FIGURE 3:**
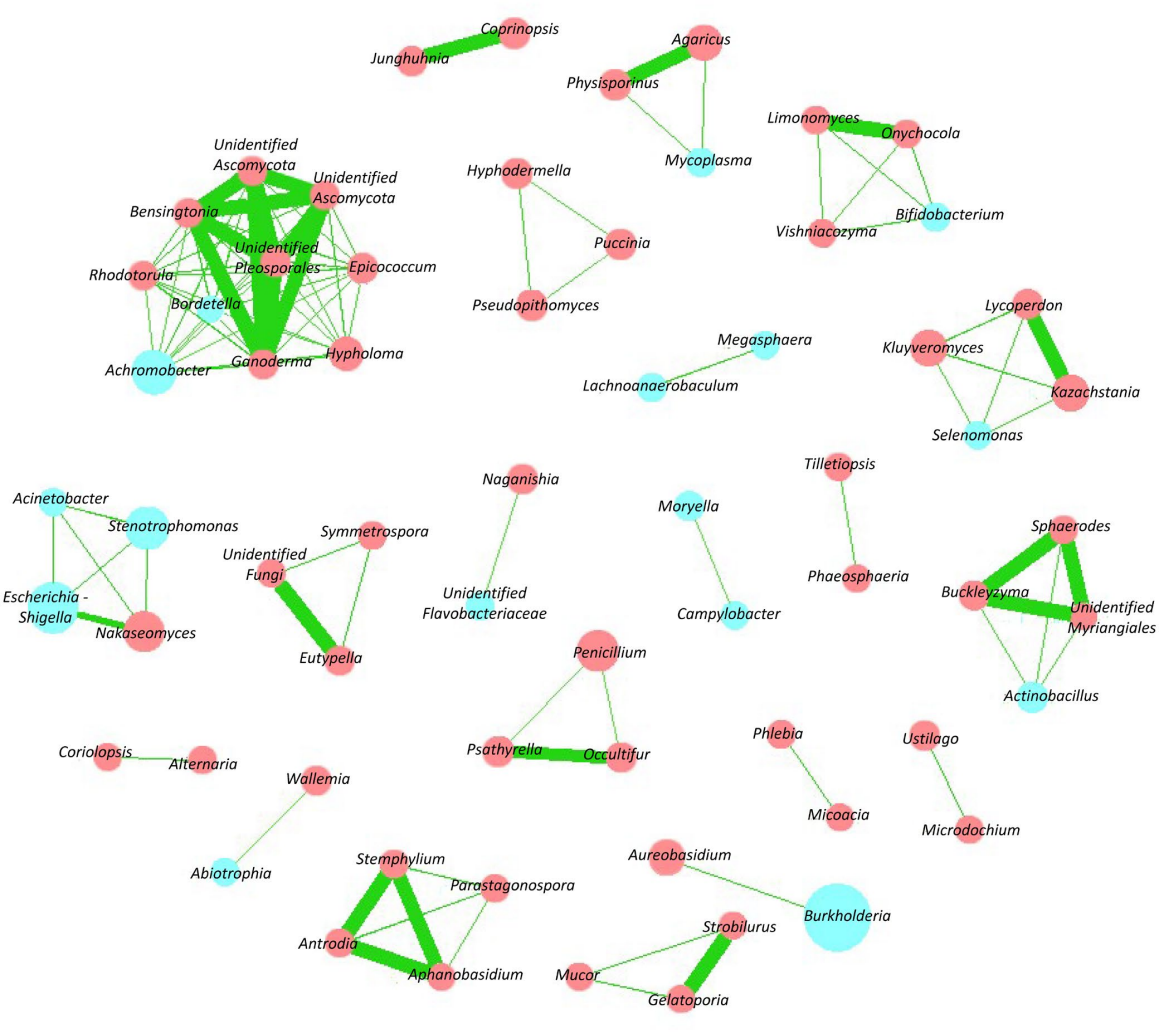
Correlation analysis based on interspecies correlation network between bacterial and fungal taxa detected in sputa (*n* = 56) of CF patients with pulmonary exacerbation (PEx). Only taxa harbouring strong intra or inter‐kingdom correlations (> 0.70) on the ReBoot method were plotted on this chart. Blue circles represent bacterial taxa, red circles the fungal taxa; circle size reflects the relative abundance of microorganisms in the dataset. Green lines connecting circles represent positive correlations (< 0.70), the thicker the line, the stronger the correlation. Taxa with < 50 reads were excluded from this analysis.

The inter‐kingdom correlation matrix, represented as a graphical network in Figure 3, displayed 63 nodes with 110 positive edges and no negative edges.

We counted six inter‐kingdom clusters and five inter‐fungal clusters with ≥ 3 nodes. All correlations included were positive (correlation coefficient > 0.7) revealing co‐occurrence patterns. The strongest (> 0.9) were between fungal taxa of low relative abundances (e.g., *Stemphylium*, *Antrodia* and *Aphanobasidium*; *Limonomyces* and *Onychocola* or *Strobiolorius* and *Gelatoporia*). Only a limited number of major CF bacteria were involved in inter‐kingdom clusters. They include a correlation between *Burkholderia* and *Aureobasidium* and *Stenotrophomonas* with *Escherichia‐Shigella*, *Acinetobacter* and *Nakaseomyces* (Figure 3).

### 3.4 Difference in Bacterial and Fungal Microbial Diversity Patterns Between the Two Centres

**FIGURE 4:**
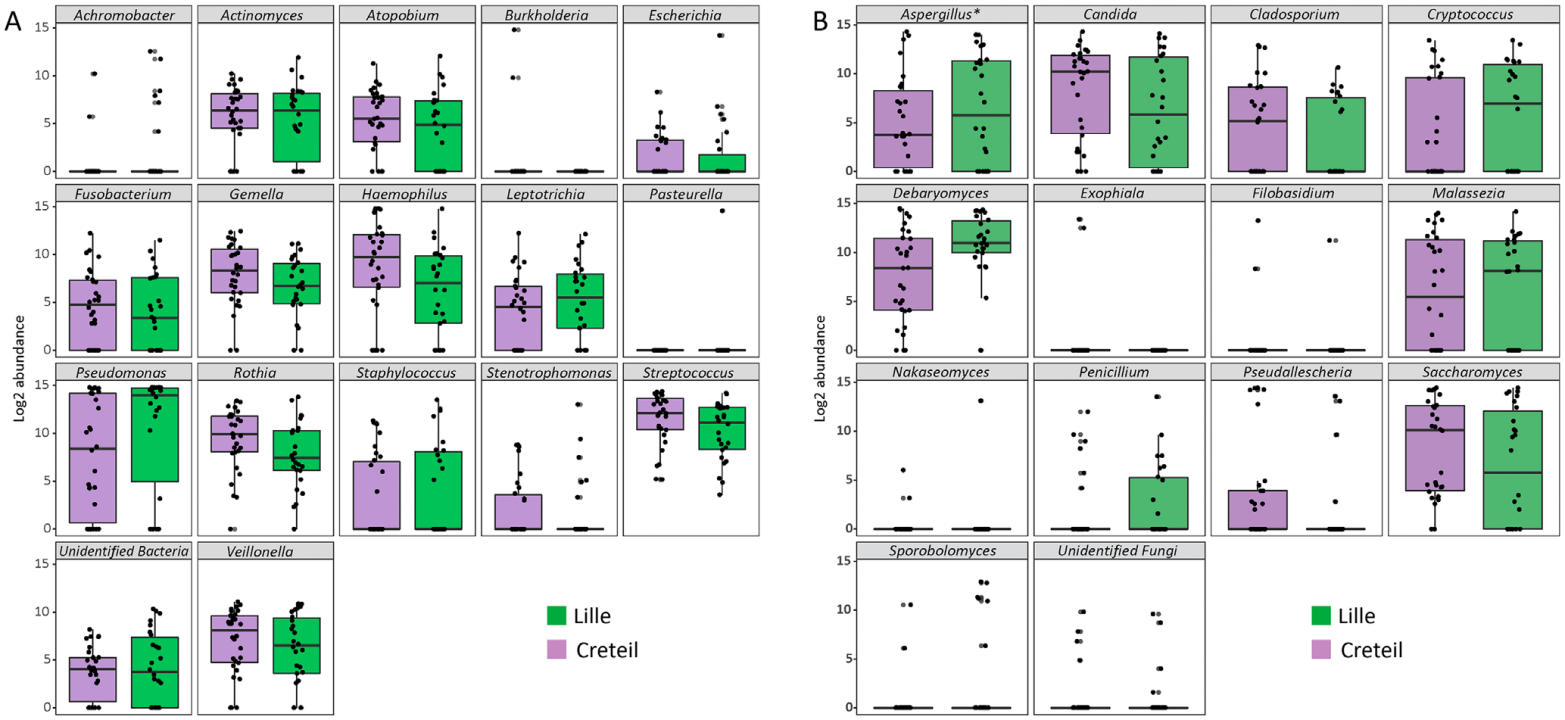
Boxplot of log2 abundances of bacterial (A) and fungal (B) taxa detected in Creteil and Lille samples. Taxa with significantly different relative abundance in Creteil versus Lille are marked with an asterisk (*). No significant differences in relative abundances of main bacterial taxa were detected. For fungi, *Aspergillus* appeared to be slightly more abundant in Lille versus Creteil (*p*‐value = 0.05).

When comparing samples from pwCF of Creteil and Lille, Shannon, Simpson and Inverse of Simpson indexes showed no differences in both bacterial and fungal alpha‐diversities (Table S3). However, PCoA indicated significant differences in bacterial microbiota beta‐diversity (*p*‐value = 0.03) between the two centres, whereas no significant difference was observed in fungal microbiota (*p*‐value = 0.23) (Figure 2B). At the taxon level, no significant difference in the relative abundances of the predominant bacteria were observed between Creteil and Lille (Figure 4). For fungi, we observed a trend towards higher *Aspergillus* abundance in Lille compared to Creteil (*p*‐value = 0.05). This trend was not confirmed by the culture results, with 13 patients with *Aspergillus* spp. in Creteil and 17 in Lille (*p*‐value = 0.09).

Analysing the inter‐kingdom correlation networks in each CRCM separately revealed a higher number of correlations in Lille (Figure S2). In Creteil, we found four small clusters (three to six nodes), two of which were inter‐kingdom; in Lille, four clusters (3 to 16 nodes), three of which were inter‐kingdom.

### 3.5 PCoA Stratified Analysis

Some demographic and medical characteristics (CF mutation, FEV1, antibiotics, etc.) were identified as potential confounding factors. To assess whether these factors might confound the association observed between CRCM and the bacterial–fungal microbiota profiles, we conducted two‐level stratified PCoA (Table 3).

**TABLE 3:** Results of principal coordinate analysis (PCoA) and Permanova tests regarding the association between bacterial or fungal microbiota patterns and centre (Creteil vs. Lille) or possible confounders (age, CF mutation, spirometry parameters, use of antibiotics or subclinical azithromycin, use of inhaled dornase alpha, bronchodilators, or steroids) analysed alone or after stratification.

	Bacterial microbiota pattern (16S)		Fungal microbiota pattern (ITS2)	
	Univariate *p*‐value^a^	Stratified *p*‐value^b^	Univariate *p*‐value^a^	Stratified *p*‐value^b^
CRCM (Creteil vs. Lille)	**0**.**03**	—	0.23	—
Age (paediatric vs. adult)	0.15	0.23	0.20	0.15
CF mutation (homozygous F508del vs. others)	0.18	0.15	0.33	0.16
FVC (above/below 60%)	0.29	0.06	0.33	0.47
FEV1 (above/below 70%)	**< 0**.**01**	**< 0**.**01**	0.12	0.18
Use of treatment within the 3 months preceding inclusion				
Subclinical azithromycin (y/n)	0.29	**< 0.01**	0.38	0.08
Systemic antibiotics (y/n)	0.21	0.17	0.71	0.41
Category of antibiotics: no antibiotics/antibiotics for *Staphylococcus* or *Haemophilus*/extended spectrum antibiotics	**0**.**01**	**0**.**03**	0.88	0.61
Inhaled dornase alpha (y/n)	0.34	0.07	0.46	0.34
Inhaled bronchodilators (y/n)	0.75	0.07	0.63	0.77
Inhaled corticosteroids (y/n)	0.15	0.10	0.89	0.05

*Note: p*‐value*s* were calculated using analysis of variance based on distance matrices (bray distance, Permanova tests). The possible confounding factors (gender, CF mutation, FEV1, FVC, use of subclinical azithromycin, systemic antibiotics, inhaled dornase alpha, bronchodilators or corticosteroids within 3‐months prior inclusion) were defined as factors associated with CRCM by a *p*‐value < 0.20. Significant results are shown in bold.

Abbreviations: CF, Cystic Fibrosis; CRCM, Centre de Ressources et de Compétences de la Mucoviscidose; FEV1, Forced expiratory volume in the first second; FVC, Forced vital capacity.

^a^ One‐level PCoA between CRCM or other factors and the profile of bacterial or fungal microbiota.

^b^ Two‐level stratified PCoA analysis.

Two‐level stratified PCoA showed a statistical association between the bacterial microbiota profile and three potential confounding factors, but no association with the fungal microbiota profile (Table 3). FEV1 (categorised as ‘below 70%’ or ‘above 70%’ [48]) and pre‐PEx systemic antibiotics (categorised as ‘no antibiotics’/‘narrow or mid‐spectrum antibiotics targeting *H. influenzae* and MSSA’/‘GNB‐ESBT and anti‐MRSA antibiotics’) were significantly associated with the bacterial microbiota profile at PEx‐time in both one‐level (*p*‐value = 0.01) and two‐level stratified PCoA (*p*‐value = 0.03) (Table 3, Figures S3 and S4). Subclinical azithromycin showed an association with the bacterial profile during two‐level stratified PCoA only (*p*‐value < 0.01, Figure S5).

## 4 Discussion

We conducted concomitant analyses using TA‐HTS to study the bacterial and fungal microbiota of pwCF during pulmonary exacerbation (PEx) in two French CRCM. Our analysis covered the taxonomic distribution and microbial diversity, as well as the intra‐ and inter‐kingdom network. Such a combined bacterial–fungal analysis in CF is rare given the < 20 articles on this theme in the literature. The results showed differences in the bacterial, not the fungal, microbiota profiles based on CRCM, and the antibiotics received within the 3 months preceding PEx.

Demographic, medical risk factors and treatments differentiating Lille's patients from Creteil's could potentially explain the differences in the bacterial microbiota profiles. Medical factors associated with CF severity (e.g., CFTR mutation) or modulating the airway microbiota (antibiotics, inhaled bronchodilators, etc.) were investigated. We observed significant differences in the distribution of the classes of CFTR mutations in Creteil and Lille (*p*‐value = 0.03) and an overrepresentation of the F508del homozygous mutation in Creteil's patients (50%), which slightly exceeding the French national rate (41%), compared to Lille's patients (17%, Table 1; [2, 48]). However, both cohorts carried mainly class I and class II mutations, both of which are associated with more severe disease [49, 50]. Overall, Creteil's patients did not have more CF‐related complications or decreased spirometry parameters compared with Lille's patients who conversely had lower FVC and FEV1. Two‐level stratified PCoA on CRCM and mutation revealed no differences. Two factors were associated with variations in the bacterial microbiota profiles in the univariate and stratified analyses: (i) FEV1 above or below 70% and (ii) pre‐PEx systemic use of antibiotics (Table 3). Francis et al. [9], in a culture study, reported that the chronic presence of *P. aeruginosa* , *S. maltophilia* or *C. albicans* unfavourably affected the evolution of FEV1. In our cohort, neither TA‐HTS nor culture did reveal significant differences in the relative abundance of these pathogens by FEV1 level. Regarding antibiotics, Creteil and Lille's patients were not exposed to the same systemic or inhaled treatments (Table 2). Overall, the differences in the bacterial microbiota profiles might be related to confounding factors such as the severity of lung damage or differences in antibiotics prescription in the two CRCM, as previously described by Thornton et al. [4]. Indeed, this author observed variations in major CF bacteria prevalence across countries, potentially related to differences in antibiotic strategies [4] and Psoter et al. [21] demonstrated the influence of meteorological elements on the acquisition of respiratory pathogens.

As already described in the literature [25, 28, 51], HTS allowed us to detect significantly more fungi than culture. The diversity of fungal taxa was increased (89 using HTS compared to 15 in culture) as well as the level of detection of main CF fungi (Figure S1). For instance, HTS allowed us to detect *Scedosporium* spp. or *Malassezia* spp. in samples with negative culture (*n* = 10 and *n* = 33, respectively). HTS revealed a higher diversity of ‘*Candida*’ yeasts (9 genera of the subphylum *Saccharomycotina*: *Debaryomyces*, *Saccharomyces*, *Candida*, *Nakaseomyces*, *Kazachstania*, *Kluyveromyces*, *Meyerozyma*, *Clavispora* and *Pichia*), whereas culture detected 4 ‘*Candida*’ species ( *C. albicans* , *Candida dubliniensis*, *Candida parapsilosis* and *Saccharomyces cerevisiae* ). HTS can therefore be an asset for detecting fungi known to be pathogenic in CF [15], but also fungi whose role has yet to be fully understood, such as *Malassezia* spp. or ‘*Candida*’ yeasts [9, 10, 24, 52, 53]. However, among respiratory uncultivable fungi of medical interest, TA‐HTS struggles to detect *P. jirovecii* and most ITS‐HTS studies do not report the presence of this fungus [25, 26, 27, 30, 31, 32, 54]. This may be due to the fact that *P. jirovecii* has only one copy of the ITS region, unlike other fungi that have multiple repeats of ITS region [55].

Fungal microbiota profiles were similar, apart from a slight increased rate of *Aspergillus* in Lille's sputa according to TA‐HTS data. In culture, the same trend was observed, and it is likely that the small sample size explains the lack of significativeness. Previous culture‐based articles reported differences in the profiles of fungi detected in pwCF's airways related to geography or meteorological factors [7, 14]. Delhaes et al. [14] observed a North–South gradient in fungi's prevalence in European countries and Van Rhijn et al. [17] demonstrated that *Aspergillus* level in the immediate environment of pwCF fluctuated according to meteorological factors. Perhaps the difference in latitude and climate between Creteil and Lille are not significant enough to strongly observe such differences. Nonetheless and given the different environmental exposure (Creteil being Paris' suburbs, hence more urbanised), we were surprised not to observe greater differences in moulds profiles, which often reflect patients' indoor environment. However, our cohort may be too small to detect this.

Up to date, few articles have analysed the airways fungal–bacterial correlations, especially in pwCF [24, 26, 27, 34, 56]. In our population, we observed six inter‐kingdom clusters (Figure 3) in which most clinically important bacterial and fungal CF pathogens were not involved. In particular, no strong correlation between *Pseudomonas* and other bacteria or fungi was found, though molecular or physical interactions have already been described (e.g., with *S. aureus* ) [57]. We observed a positive correlation between *Nakaseomyces* (*Nakaseomyces glabratus* also known as *Candida glabrata*) and *Stenotrophomonas*, an important CF bacterium (Figure 3) and another between *Burkholderia* and *Aureobasidium*. However, these strong correlations, were only found in one patient each. To our knowledge, such interactions have not been described before and neither *N. glabratus* nor *Aureobasidium* are known to be clinically important CF fungi. However, Granchelli et al. [ 5], in a culture‐based study, found a possible association between ‘*Candida*’ yeasts and *Stenotrophomonas*, with a slightly higher risk of future ‘*Candida*’ infection when pwCF carried *S. maltophilia* . This study did not specify the species of ‘*Candida*’ and did not mention *N. glabratus* . The authors also indicated that *Burkholderia* spp. was associated with a lower likelihood of future fungal infection, but no association with *Aureobasidium* sp. was described. Other correlations were observed in only one CRCM, for example, a positive correlation between *Aspergillus* and *Achromobacter* in Creteil (Figure S2), which also complies with what Granchelli et al. found [5]. It remains unclear whether these differences in the inter‐kingdom correlations detected in Creteil and Lille were due to confounding factors or to the small groups we had. Soret et al. [27] also analysed the inter‐kingdom crosstalk between fungi and bacteria in 33 pwCF. Unlike us, they found three large bacterial–fungal clusters involving important fungi: *Scedosporium* was positively correlated with nine bacterial taxa, *Candida* was negatively correlated with four bacterial taxa, and *Aspergillus* had four positive and one negative correlations with bacterial taxa. Liu et al., in a study on 116 asthmatic patients, observed correlations between *Aspergillus* or *Candida* and different bacterial taxa (such as *Prevotella* or *Leptotrichia*) [56]. At this point, data on inter‐kingdom crosstalk are still scarce in chronic respiratory diseases, especially in CF and further studies on larger populations are warranted to refine the role of these interactions within the Climax or Attack communities described in pwCF [27].

The main strength of our study is the concomitant analysis of fungal and bacterial microbiota using TA‐HTS in pwCF from two French centres. To date, few studies have performed this type of analysis [25, 27], which seems as an important prerequisite to understand the pathophysiology and the interactions between commensal and pathogenic microorganisms in CF. The main limitation of our study is inherent to its small size, which may have impaired its statistical power. Given the bicentric design, sputum collection was not fully standardised, which may have influenced its results. For fungal cultures, it would have been interesting to add additional media to those routinely used in the participating CRCM (Sabouraud‐Gentamicine‐Chloramphenicol and CHROMagar *Candida*) in order to improve the detection rate of certain fungi more difficult to culture, such as *Exophiala* or *Scedosporium*. Another limitation is inherent to the short‐read TA‐HTS technique which does not detect all types of bacteria or fungi, for example, non‐tuberculous mycobacteria or *Pneumocystis jirovecii*. Further studies using long‐read TA‐HTS or shotgun metagenomics could help assess the bacterial–fungal microbiota with more accuracy [58, 59]. Eventually, our patients were included before the era of CFTR modulators [57].

In conclusion, we analysed the diversity of bacterial and fungal communities of pwCF and the intra‐ and inter‐kingdom correlations during PEx according to different medical or environmental criteria. We observed inter‐centre differences in the bacterial microbiota profiles related to pre‐PEx antibiotics intake and lung damage severity; no differences concerning the fungal microbiota. The inter‐kingdom correlations we found between bacterial and fungal taxa warrant further investigation in larger cohorts to unveil potential physical or molecular interactions and appreciate their impact on microorganisms themselves and the host's inflammatory response. Future studies should compare CF patients' microbiota before and after the introduction of CFTR modulators, which are likely to induce substantial changes in microbiota profile and in potential microbial interactions that can lead to biofilm formations in patients' airways.

## Author Contributions

**Cécile Angebault:** investigation, writing – original draft, writing – review and editing, visualization, formal analysis, data curation, methodology, validation. **Louise‐Eva Vandenborght:** formal analysis, visualization, resources, writing – review and editing. **Laurence Bassinet:** resources, writing – review and editing, investigation. **Nathalie Wizla:** writing – review and editing, resources, investigation. **Agnès Ferroni:** resources, writing – review and editing. **Rodrigue Dessein:** writing – review and editing, resources, investigation. **Natacha Remus:** resources, writing – review and editing, investigation. **Caroline Thumerelle:** writing – review and editing, resources, investigation. **Nathalie Fauchet:** resources, writing – review and editing, investigation. **Ralph Epaud:** writing – review and editing, resources, investigation. **Laurence Delhaes:** visualization, writing – review and editing, formal analysis, investigation, methodology, project administration. **Françoise Botterel:** conceptualization, investigation, validation, visualization, writing – review and editing, writing – original draft, funding acquisition, supervision, formal analysis, project administration, methodology.

## Consent

The authors testify that all procedures contributing to this work complied with the ethical standards of the Helsinki Declaration of 1975, as revised in 2008. This work was part of the MucoBacMyco project, which was approved by an *ad hoc* Ethical Committee named *Comité de Protection des Personnes d'Ile de France IX* (N° CPP‐IDF IX‐12‐011). All patients received full information on the research project from their physicians before being asked to participate. If they agreed to participate, they would sign informed consent prior to inclusion. For children, both parents were informed and then signed the informed consent. All included patients were attending their CRCM for normal CF visits associated with PEx and no sample was specifically drawn for the MucoBacMyco study, only routine samples were analysed for the project.

## Conflicts of Interest

Over the past 5 years, F.B. has received grants from Astellas, payments for lectures from Mundipharma and Gilead and travel expenses from Pfizer, Mundipharma and Gilead. C.A. received congress grants from Gilead and Biosynex and a payment for a lecture from Pfizer. R.E. received consulting fees, congress grants and payments for lectures from GSK and Astra Zeneca and is on the advisory board of Astra Zeneca, Novartis and Sanofi. R.D. received payments for lectures from Shionogi and Beckton Dickinson. C.T. received travel and congress grants from GSK and Sanofi. L.D. received payment for a lecture from Gilead and travel/congress grants from Gilead and Viatris Medical. The other authors declare no conflicts of interest.

## Supporting information


Data S1.

## Data Availability

The data that support the findings of this study are openly available in Genbank SRA at https://www.ncbi.nlm.nih.gov/sra/PRJNA1011866, reference number PRJNA1011866.
