# Supplementary material for: Airway Mycobiota—Microbiota During Pulmonary Exacerbation of Cystic Fibrosis Patients: A Culture and Targeted Sequencing Study
Source: Mycoses. 2025 Jan 16;68(1):e70024. doi: 10.1111/myc.70024 (PMC11736540; doi:10.1111/myc.70024)

**APPENDICES – SUPPLEMENTARY DATA**

**Table S1.** **List of demographics, past and current medical history, and treatments data collected in the case report form (CRF) of included patients.**

**
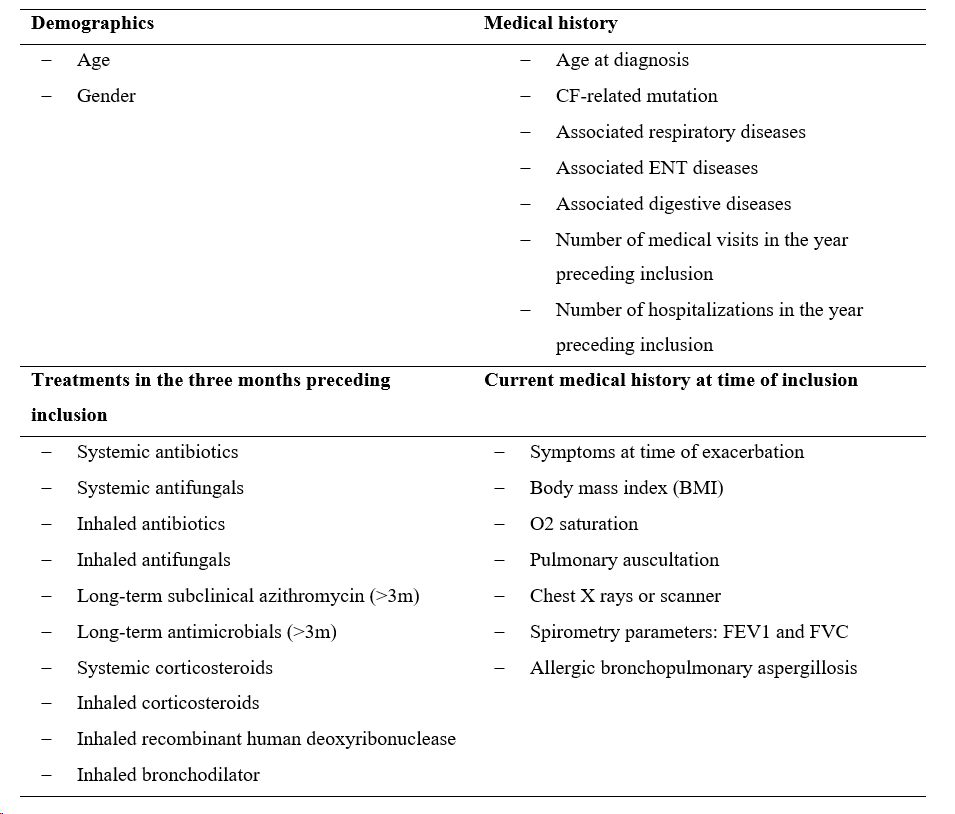
**

**Table S2. Detailed description of extended spectrum bi/tritherapy targeting Gram-negative bacteria (GNB-ESBT) in the year prior to the pulmonary exacerbation (PEx).**

| **Description of GNB-ESBT combinations** | **Creteil, n=30** | **Lille, n=26** |
| --- | --- | --- |
| Meropenem + Tobramycin | 2 | - |
| Meropenem + Co-trimoxazole + Tobramycin | 1 | - |
| Meropenem + Colistine | 1 | - |
| Meropenem + Ciprofloxacin | - | 1 |
| Ceftazidim + Tobramycin | 1 | 1 |
| Ceftazidim + Co-trimoxazole | 1 | - |
| Piperacillin/Tazobactam-Amikacin | 1 | - |
| Piperacillin/Tazobactam-Ciprofloxacin | 1 | - |
| Co-trimoxazole + Amoxicillin/Clavulanate | - | 1 |
| Aztreonam + Amoxicillin/Clavulanate | 1 | - |
| Aztreonam + Tobramycin | - | 1 |
| Aztreonam + Ceftazidim | - | 1 |
| Ceftriaxone + Levofloxacin | - | 1 |
| Ticarcillin/Clavulanate+ Ciprofloxacin | - | 1 |
| Ticarcillin/Clavulanate + Colistine + Aztreonam | - | 1 |

**Table S3. Alpha diversity measurements (Shannon, Simpson, and Inverse Simpson indexes) for bacterial (A) and fungal (B) metagenomic analyses performed on 56 sputa of pwCF at exacerbation time (30 patients from Creteil CF cohort and 26 from Lille).**

|  | **Creteil** | | **Lille** | |
| --- | --- | --- | --- | --- |
| A - Bacterial diversity at genus level | | |  |  |
| Shannon | 1,06 | [0,81-1,31] | 0,97 | [0,66-1,25] |
| Simpson | 0,44 | [0,33-0,54] | 0,4 | [0,28-0,52] |
| Inv. Simpson | 2,43 | [1,90-2,97] | 2,55 | [1,74-3,36] |
| B - Fungal diversity at species/section level | | |  |  |
| Shannon | 0,94 | [0,74-1,14] | 1,07 | [0,89-1,26] |
| Simpson | 0,46 | [0,37-0,55] | 0,52 | [0,44-0,60] |
| Inv. Simpson | 2,45 | [1,87-3,03] | 2,56 | [2,03-3,08] |

**Figure S1. Histogram of the concordance between culture and high-throughput sequencing (HTS) for the main fungal (A) and bacterial (B) taxa in the respiratory mycobiome of cystic fibrosis (CF) patients.** The lower histogram represents the number of positive samples for every microorganism whatever the technique used (culture and/or HTS).


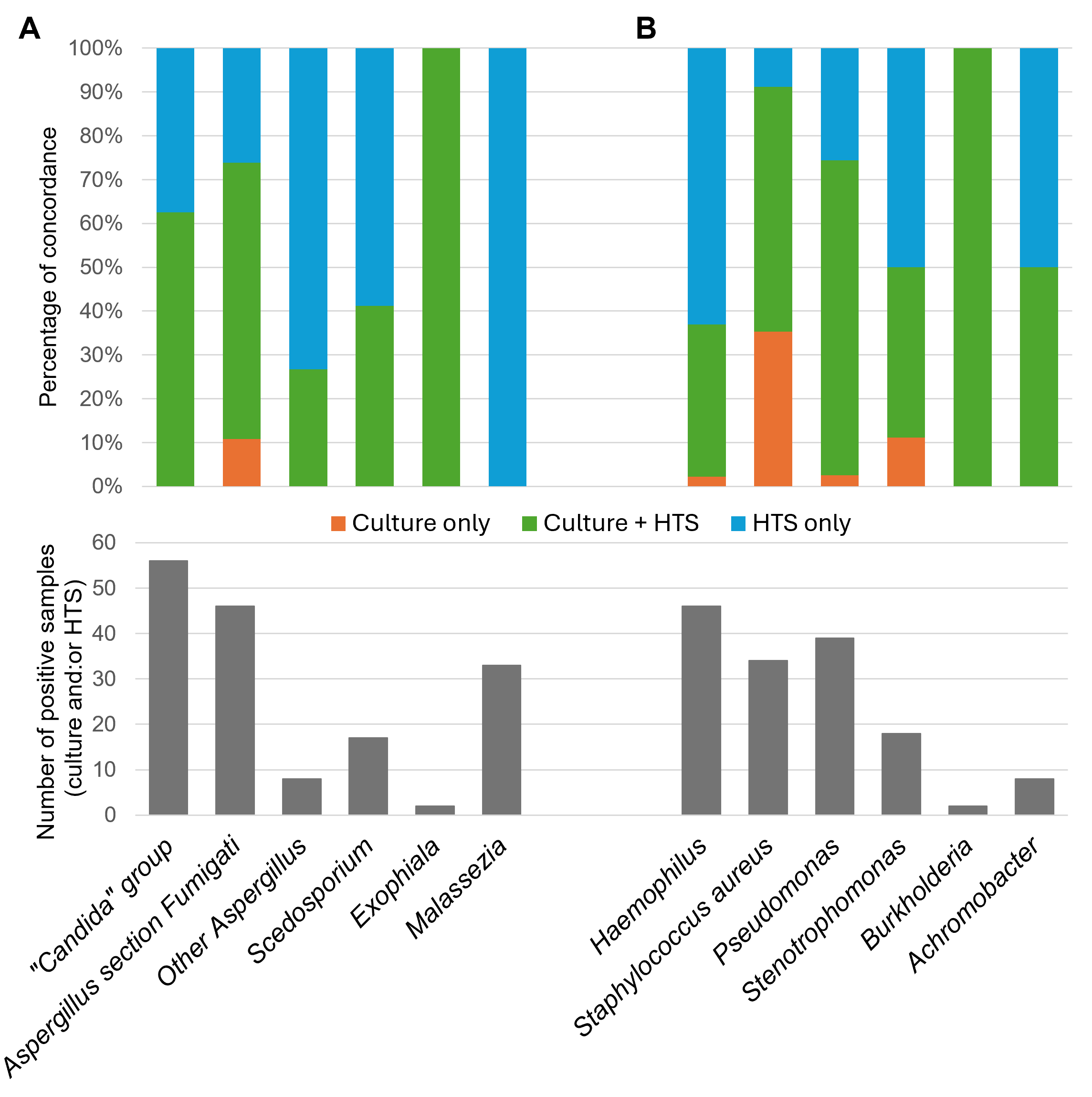


**Figure S2. Inter-kingdom co-occurrence networks between bacterial and fungal taxa detected in 26 CF patients from Creteil and 30 from Lille (ReBoot method).** Bacteria are represented by blue circles while Fungi are in red. The circles size is associated with relative abundance of microorganisms in the dataset. Green lines connecting circles represent strong positive correlations (>0,7). Larger lines are associated with increased correlation strength. Taxa with less than 50 reads were not included.


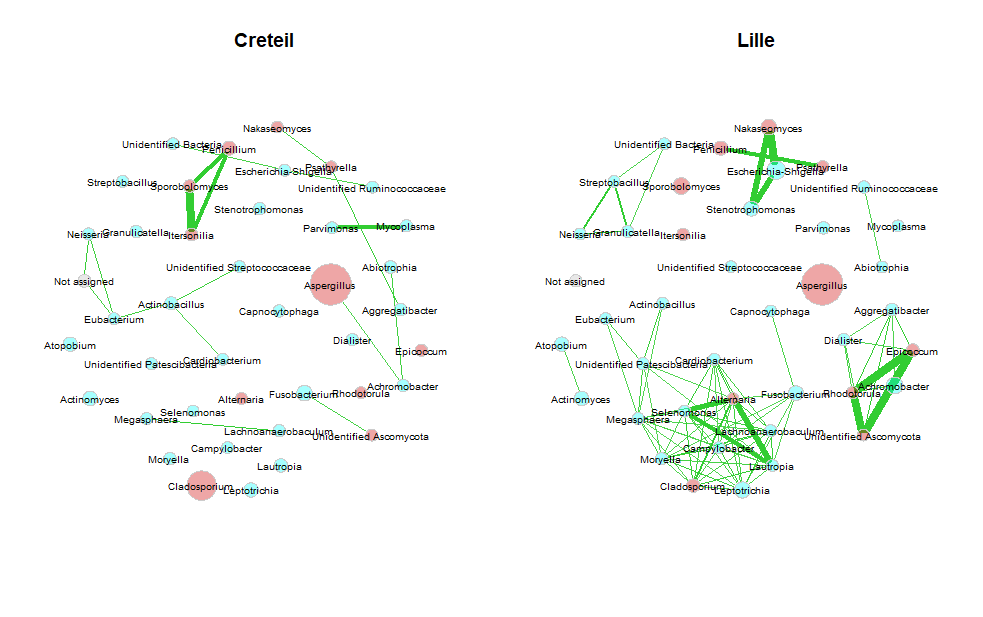


**Figure S3. Diversity of bacterial microbiota profiles according to FEV1 (above or below 70%) alone or stratified on CF center (Creteil or Lille) assessed through PCoA analysis.** Statistical significance was assessed through Permanova test.


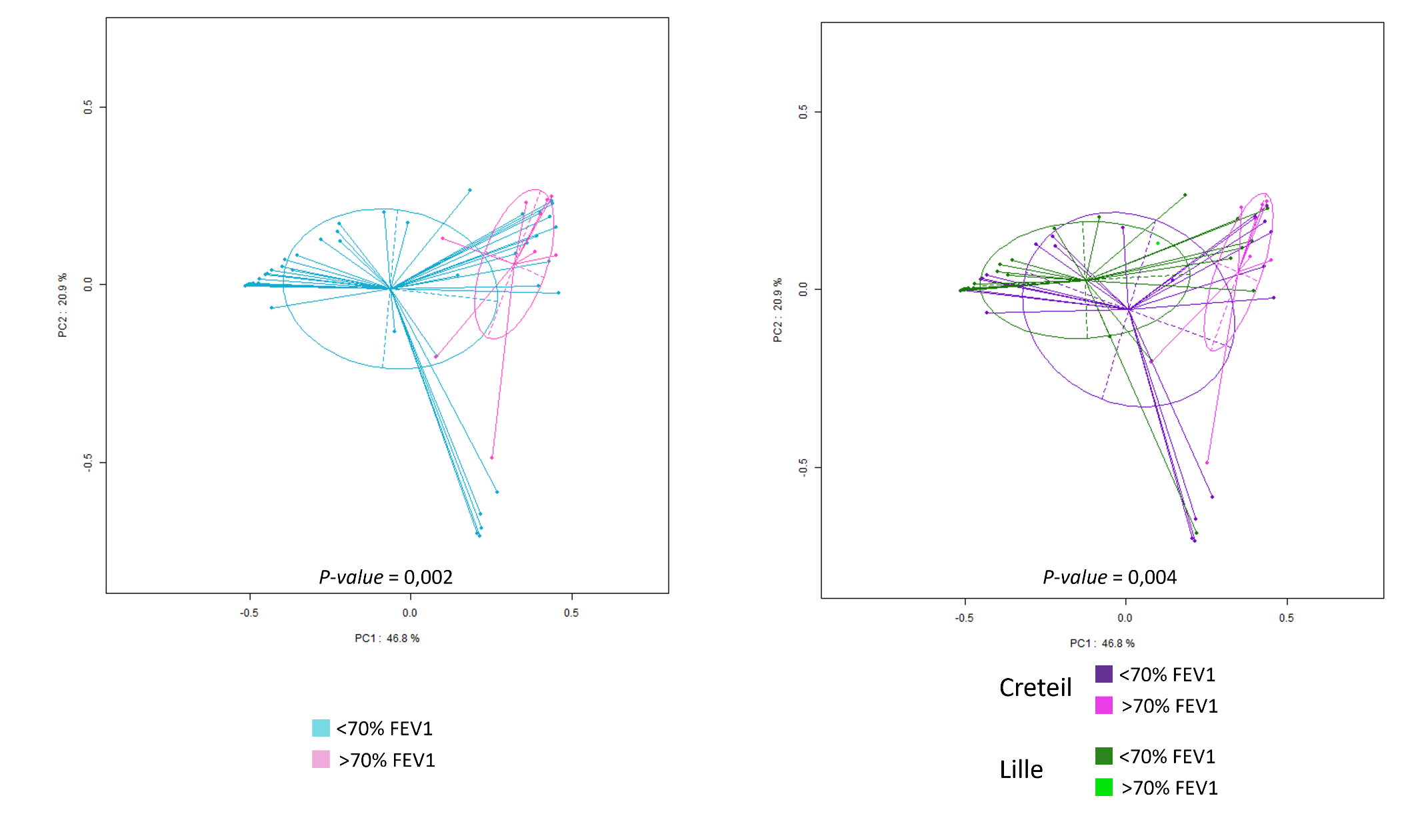


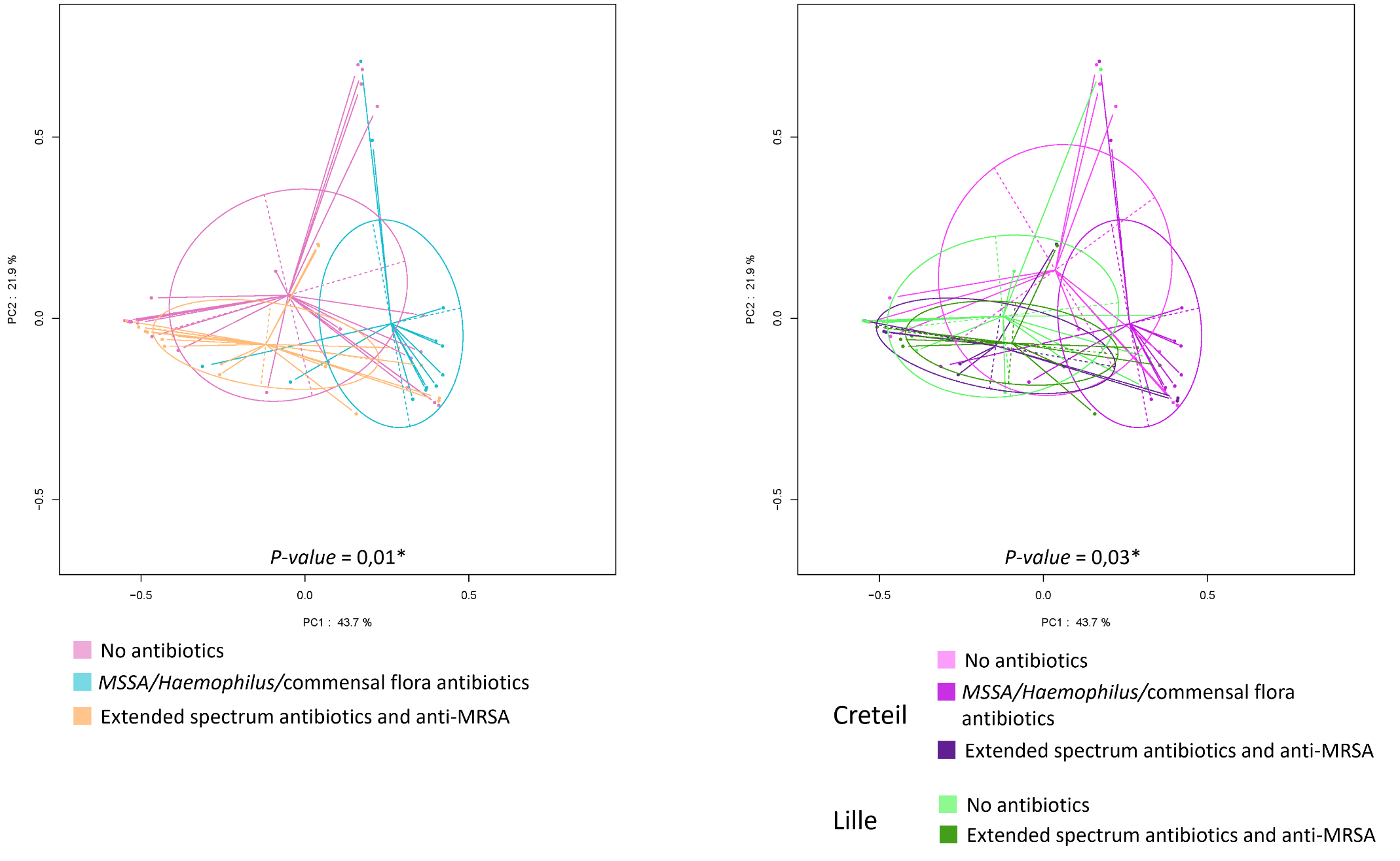
**Figure S4. Diversity of bacterial microbiota profile according to category of systemic antibiotics used within 3-months priori inclusion alone or or stratified on CF center (Creteil or Lille) assessed through PCoA analysis.** Statistical significance was assessed through Permanova test. The antibiotics were categorized as *(i)* no antibiotics; *(ii)* narrow or mid-spectrum antibiotics targeting MSSA, *Haemophilus* or other bacteria from commensal flora and *(iii)* extended spectrum antibiotics and anti-MRSA.

**Figure S5. Diversity of bacterial microbiota profile according to intake of subclinical azithromycin within 3-months priori inclusion alone or stratified on CF center (Creteil or Lille) assessed through PCoA analysis.** Statistical significance was assessed throught Permanova test.


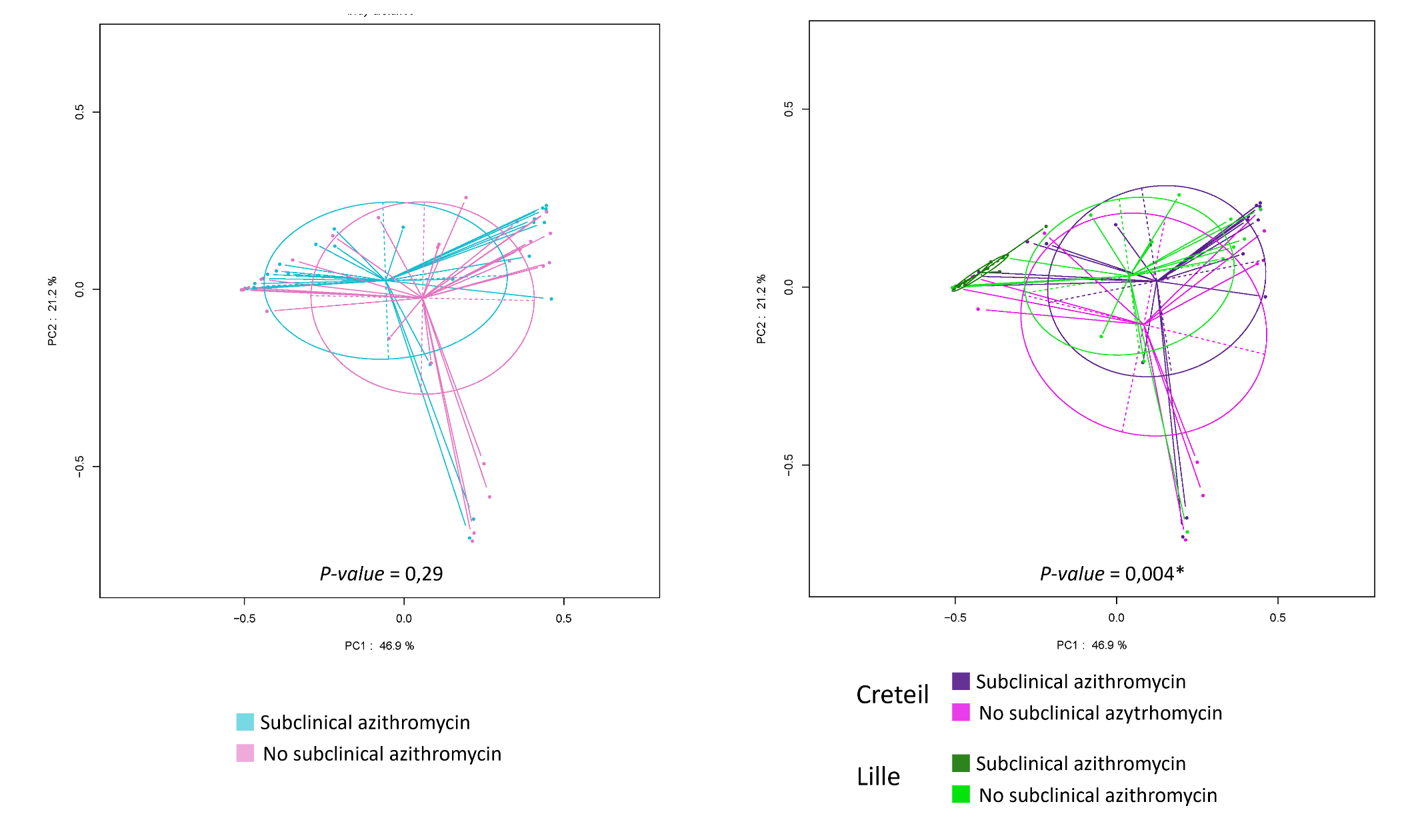

Supplement: Supplementary file 1 — Data S1. [file MYC-68-e70024-s001.docx]
